# The Voluntary Hospitals' Outlook

**Published:** 1914-01-03

**Authors:** 


### The Hospital

The Workers' Newspaper of Administrative Medicine and Institutional Life, Administration, National Insurance and Health.

No. 436, Vol. LV. SATURDAY, JANUARY 3, 1914. PRICE ONE PENNY

### THE VOLUNTARY HOSPITALS' OUTLOOK.

What is the outlook for the voluntary hospitals in 1914 and thenceforward? At the beginning of 1913, with the introduction of National Insurance, there was reason for thoughtful people to become anxious in regard to the future of the voluntary hospitals. There can be no doubt that the financial position of these institutions has been affected to a small degree by the unpopularity of National Insurance and the fears of overburdening taxation arising out of it. The repulse of the medical profession in its encounter with the Chancellor of the Exchequer did infinite mischief. The difficulties of introducing a system of National Insurance should never have been permitted to degenerate into a warfare, which was conducted as a struggle for supremacy between ambitious politicians and the members of a learned profession. The besting of the practitioners was brought about by feeble leadership on the one hand, and unworthy tactics and falsified facts on the other. The managers of the voluntary hospitals had the wisdom to stand aloof from this regrettable conflict. They so have succeeded in eliminating an increasing number of trivial cases from all claim to hospital treatment, whilst they have opened their doors freely to insured persons needing admission as in-patients. By this policy the voluntary hospitals are, in fact, at the moment the main financial foundation by which National Insurance, on an actuarial basis, is kept from liquidation. This position cannot continue indefinitely, and before, or at the latest, by the autumn of 1915, a new basis for providing in-patient hospital care for all insured persons will have to be found.

Our view is, and always has been, that that basis should be a *pro rata* system, by which contracts should be entered into between the hospitals and the approved societies as in Germany, or between the hospitals and the Insurance Commissioners representing the Treasury, whereby a sum equal to the *pro rata* cost of treating every insured person in a voluntary hospital should be paid to each institution out of the funds that stand to the credit of National Insurance. This progressive change should be accompanied by a plan whereby adequate hospital provision would be made for the reception and treatment of paying cases, with those classes of the community which are not insured but which can afford to pay the whole, or some portion, of the cost of their treatment as in-patients.

The system upon which this can readily be done would not entail any permanent charge upon State funds or the pockets of the rate and tax payers. Such a system is at present widely prevalent in the United States and various countries throughout Europe. The demand for such hospital accommodation in these islands is growing every day. Its need is admitted by politicians and an increasing number of the leaders of both political parties and by public opinion outside politics altogether. Once these changes, which, though important, will be found simple of accomplishment, are effected, our voluntary hospital system will enter upon a career of increased usefulness. For it will rest upon financial foundations strong enough to guarantee the permanence of this system.

We have had striking evidence in the last month that the voluntary system is not only not dead but more deeply centred in the hearts of the British people than ever before. The magnificent sum of nearly half a million sterling, permanently invested through the King's Hospital Fund for the benefit of the Metropolitan voluntary hospitals, means much. It teaches that an active propaganda in favour of the voluntary principle, systematically carried on twenty years ago amongst the leading financiers and business men in the City of London, has taken deep root in the hearts of these hard-headed and successful leaders of commerce, who became convinced that the voluntary hospital system is a precious national asset which must be preserved and strengthened.

Sir Julius Wernher and his firm, when they first became merchant princes in the City of London, had never given deep thought to the responsibilities of wealth and the privilege of personal service in the cause of the sick. The head of the firm, Sir Julius Wernher, came, after deep consideration, to a knowledge of what these principles mean to the great working and industrial classes of these islands.

He took upon himself personal service, a course which is precious to those who knew the facts, as indicating that millionaires are often men of sterling heart and deep sympathies. A number of other merchant princes, notably the members of the house of Rothschild, the old firm of J. S. Morgan and Co., Baring Brothers and Co., Hambro and Sons, and a number of others, together with individual philanthropists like Lord Mount Stephen, Lord Strathcona, Sir Ernest Cassel, the late Mr. J. G. Lambert are a few only of the many who might be mentioned as remarkable for their adherence to and belief in the voluntary hospital service.

There are not wanting indications that the time is approaching when the combined efforts of wealthy men in this country will place the voluntary hospitals on an adequate financial foundation. Adequate because so long as the voluntary hospitals continue to maintain the highest standard of efficiency, the people for whose protection and comfort they provide will readily raise the annual gifts from the multitude of small givers to a sum sufficient for their maintenance and extension. It is a remarkable and encouraging fact that as 1913 is drawing to a close the late Mr. Charles Ansell's will should yield £200,000 to the Metropolitan hospitals. Mr. Ansell was well known to the writer. He came forward spontaneously seeking advice, and wished for opportunities to contribute liberally to the voluntary hospitals. He was ever ready to give personal service in their cause. He was won to take an interest in these institutions by the example set by Sir Julius Wernher, to whose firm Mr. Ansell's firm acted as brokers. Evidence is accumulating everywhere of the power of attraction which the voluntary system and that of personal service has for the wealthiest of mankind. The outlook for the voluntary hospitals is, therefore, most encouraging. Providing younger men can be found to take the place of those who preceded them in the City of London, and to follow the example of the merchants we have mentioned, it will prove good for the merchant princes and for the hospitals.

